# The extent to which medical specialists provide Clinical Work-Integrating Care (CWIC) and their perceived role-responsibility: a mixed-methods study

**DOI:** 10.1186/s12913-024-12137-y

**Published:** 2025-03-27

**Authors:** Lana Kluit, Astrid de Wind, Annechien Beumer, Coen A. M. van Bennekom, Angela G. E. M. de Boer

**Affiliations:** 1 Amsterdam UMC Location AMC, University of Amsterdam, Department of Public and Occupational Health, Meibergdreef 9, 1105 AZ Amsterdam, the Netherlands; 2 Amsterdam Public Health research institute, Societal Participation and Health, Amsterdam, the Netherlands; 3 Amphia Hospital, Upper Limb Unit Department of Orthopedic Surgery, Breda, the Netherlands; 4 Heliomare Rehabilitation Centre, Research and Development, Wijk aan Zee, the Netherlands; 5 Cancer Center Amsterdam, Cancer Treatment and Quality of Life, Amsterdam, the Netherlands

**Keywords:** Medical specialists, work participation, Return to work, Occupational health, Physicians' practice pattern, Integrated health care systems, Mixed-methods study

## Abstract

**Background:**

Awareness among medical specialists about patient work concerns is important because work and health are linked. In Clinical Work-Integrating Care (CWIC), specialists adopt the notion that work can affect health, and medical actions can affect work participation, and they act according to that notion. This study aims to assess the extent to which specialists provide CWIC and to obtain perceptions of medical specialists about their professional role-responsibility in providing CWIC.

**Methods:**

This cross-sectional mixed-methods study involved quantitative questionnaires and qualitative interviews with medical specialists. The self-developed 18-item questionnaire evaluated the extent and type of CWIC provision (rating scale 0–4; Never = 0 to Always = 4) and how role-responsibility was perceived, while the interviews offered more in-depth insights. Descriptive statistics for the questionnaire data and thematic analyses for the interview data were applied.

**Results:**

We attained 160 questionnaires (female 64%, 93% non-surgical specialists) and 11 interviews (female 64%, 91% non-surgical specialists). Specialists often asked patients about work (mean score 3.1), sometimes about work history (mean score 2.2) and the conversation about work was usually started by the specialist (mean score 2.9). Conversations about work often concerned the influence of work on disease (2.4) and the influence of disease (2.5) or treatment (2.1) on work ability, but rarely about the legal aspects related to sick leave (1.5). The specialists' perceived role-responsibility was summarized in three themes: 1) understanding that work and health (problems) are linked including asking patients about work and investigating work factors, 2) supporting work participation within a specialist’s expertise including focus on patients’ health and prevention of sick leave, and 3) possibilities and limitations of the healthcare system including work participation as treatment goal and cooperation with occupational health care.

**Conclusions:**

Medical specialists in our survey usually asked about patients' work, but they often did not take a work history. Limitations within the healthcare system hinder comprehensive work-integrating support by specialists, defining the boundaries of CWIC to within hospital care.

**Supplementary Information:**

The online version contains supplementary material available at 10.1186/s12913-024-12137-y.

## Background

Paying attention to the work participation issues of patients in a clinical context is important, because work and health are inextricably linked [1]. Work-related factors can be (partly) responsible for the development or exacerbation of a disease, but work can also have a positive influence on health [2]. Furthermore, disease or its accompanying treatment can affect work ability both positively and negatively [3]. In Clinical Work-Integrating Care (CWIC), healthcare professionals have the understanding that work-related factors can affect health, and medical actions can affect work participation [4]. A healthcare professional who acts according to the concept of CWIC addresses work during patient consultations, which can be as limited as asking “do you work?” or as broad as actively supporting patients to remain at work when they wish to do so. By actively inquiring about work, medical specialists incorporate both the beneficial and harmful effects of work participation into their diagnostics as well as their treatment plan, thereby potentially enhancing the patient’s health, improving the quality of care, and increasing work participation for individuals with health problems.

A medical specialist’s awareness of a patient’s work participation concerns is furthermore important because access to occupational health care is not guaranteed for many patients. Worldwide, 75.2% of economically active workers do not have this access [5]. Furthermore, vulnerable groups, such as self-employed persons, students, or informal workers, are often not covered by work accident insurance systems [6]. This results in a significant group of (potential) workers not having access to occupational healthcare. To enhance the well-being of all workers, especially in vulnerable groups, medical specialists could act from their position as health advocates by being aware of the work-related health (problems) their patients have [7, 8]. To support their patients, they could use their medical knowledge to positively contribute to patient work participation needs. One way in which this could be accomplished is by providing CWIC.

In current practice, medical specialists provide CWIC to varying extents, from asking about a patient’s occupation to extensive patient-physician collaboration with regard to work-related problems [9]. In many countries, discussing work-related concerns is embedded within legislation [9]. However, this legislation usually focuses on issuing sick notes or facilitating rehabilitation following a work-related accident [9]. This often does not address the patients’ ongoing support needs for obtaining practical tools to manage their disease or disability in relation to their work [10–13]. Thus, by not exploring work concerns during clinical encounters, patients are deprived of the important expertise medical specialists could offer in relation to work and health support.

In our previous research, patients expressed four categories of work-related needs in the context of CWIC that they wished to be able to address together with their medical specialist: 1) does work influence disease, 2) how to maintain work ability despite disease, 3) how to accept loss of work ability due to disease, and, finally, 4) what regulations apply [4]. It is unknown, however, the extent to which medical specialists in the Netherlands provide CWIC and address these work-related needs with patients. Furthermore, the question arises as to what medical specialists perceive their professional role-responsibility to be in facilitating their patients’ needs [14, 15]. Therefore, the aims of this study are (i) to determine to what extent medical specialists provide CWIC in current clinical practice, and (ii) to obtain perceptions of medical specialists about their professional role-responsibility in providing CWIC.

## Methods

### Study design

A cross-sectional mixed-methods study design was used with a two-step approach. Firstly, data was gathered through an anonymous web-based questionnaire using an online questionnaire tool (i.e., Lime survey) during the period of February–June 2022. In the next step, interviews with individual medical specialists were conducted in May–June 2022 to gain further understanding into the results of the questionnaire by exploring the medical specialists’ perceptions of their role-responsibility. We used the GRAMMS guidance for reporting this mixed-methods study [16].

Using a mixed-methods study design strengthened our research by integrating quantitative and qualitative data sources [17]. We commenced the research process with a questionnaire to quantify the extent of provision of CWIC and to quantify the opinions concerning the specialists’ role-responsibility in CWIC held by an ample number of medical specialists. Next, we delved deeper into these quantitative findings by analyzing the perceptions of medical specialists as expressed in interviews. This qualitative analysis involved an iterative process, offering the advantage of attaining a comprehensive understanding of CWIC in Dutch clinical practice [18, 19].

### Setting

Legislation in the Netherlands has a structure that separates clinical healthcare from occupational healthcare [20]. Within this structure, the responsibility for assessing work ability, issuing a medical certificate, and guiding sick workers solely belongs to occupational healthcare professionals [21]. Medical specialists working within clinical healthcare are not allowed to issue a medical certificate for patients with whom they have a treatment relationship [22]. This has led to the impression that work has no place within clinical healthcare [23, 24]. However, many Dutch (potential) workers do not have access to occupational health care [23]. Thus, a healthcare gap exists for Dutch workers who become ill [23, 25–27].

### Study sample and recruitment

The web-based questionnaire aimed to reach medical specialists and physicians in postgraduate specialty training within the Netherlands. Participants in this study were eligible if they met the following inclusion criteria: being a medical specialist or physician in postgraduate specialty training practicing in secondary or tertiary healthcare in a hospital or rehabilitation clinic. Participants were excluded from this study based on the following criteria: medical specialists who (i) did not treat patients within working age (18–67 years) (i.e., geriatricians and pediatricians), (ii) did not have outpatient clinical consultations involving patients (i.e., anesthetists, medical microbiologists, pathologists, and radiologists), or (iii) worked in primary or occupational health care (i.e., general physicians and occupational health physicians).

Three strategies were used to disseminate the questionnaire. Firstly, dissemination was executed through the newsletter of several medical specialist associations. Secondly, we contacted medical specialists within our own networks directly and asked them to disseminate our questionnaire (i.e., snowballing). Thirdly, the questionnaire was spread through a social media campaign on LinkedIn, which is commonly used by healthcare professionals in the Netherlands. At the end of the questionnaire, participants could express their interest in participating in an interview (including their contact information), and all those who did (*n* = 22) were later contacted by one of the researchers. Of the interested specialists, eleven responded to our invitation and interviews were scheduled. With regard to rehabilitation specialists, seven were interested in an interview, but we stopped planning interviews after data saturation was reached, which was after with five interviews within this subgroup. Furthermore, after additional six interviews with non-rehabilitation specialists, data saturation was reached.

### Quantitative measures

The questionnaire (Supplementary Material 1) was based on our previous research in which patients’ perspectives and needs regarding CWIC were explored [4]. Before the questionnaire was disseminated, a pilot test was performed with ten medical specialists to determine the face validity of the questions. Minor revisions of the questions were made based on the feedback of the respondents. The questionnaire consisted of three clusters of questions: 1) background characteristics, 2) extent to which medical specialist provide CWIC in their current practice, and 3) perceptions of the role-responsibility of different components of CWIC.

The following background characteristics were determined: medical specialization (e.g., cardiology, dermatology, gastroenterology), years of medical work experience (i.e., < 5, 5–10, 10–20, and > 20), gender, estimation of the percentage of consultations with working patients (i.e., 0–20%, 20–40%, 40–60%, 60–80%, 80–100%, and unknown), and type of patients (i.e., mostly chronic or mostly acute conditions).

The extent to which medical specialists provide CWIC in clinical practice was determined with 18 questions of which 16 could be answered on a five-point Likert scale from never to always (0 = never, 4 = always). Six questions asked participants to rate how often the topic of work was discussed and on whose initiative (e.g., how often do you ask whether your patient works?). Four questions asked them to rate how often different work-related topics were discussed (e.g., influence of work on disease). Six questions asked on whose initiative contact with an occupational healthcare provider was established (e.g., in case of contact with an occupational health physician, how often was this on the request of your patient?). Two categorical questions asked about the means through which contact was established with an occupational healthcare provider (i.e., in writing, by telephone, or by other means).

The perceptions of the medical specialist’s professional role-responsibility were measured with 11 questions which could be answered on a five-point Likert scale (1 = strongly disagree, 3 = neutral, 5 = strongly agree) (e.g., ‘Do you agree that giving advice about balancing disease and work participation is part of your responsibility?’).

### Qualitative data collection

Three main topics were addressed during the interviews: (i) general opinion about CWIC, (ii) cooperation with an occupational health physician, and (iii) the specialist’s perception of his or her professional role-responsibility (Supplementary Material 2). With regard to the last topic, the four groups of patients’ work-related concerns from our previous study were addressed: does work influence disease, how to maintain work ability, how to accept loss of work ability, and what regulations are involved [4]. The most notable results from the questionnaire (determined through exploring of the preliminary results) were presented during the interviews, and the medical specialists were asked to reflect on these results. Before each interview was conducted, background characteristics were gathered (i.e., medical specialization, years of medical experience, gender, and whether the interviewee held special expertise in the area of health and work).

All interviews were held online and recorded using Microsoft Teams [28]. All interviews were conducted by LK (MD, researcher, female, trained and experienced in qualitative research) or a research assistant (BSc, male, trained in qualitative research), while the other acted as observer. The observer took notes during the interview, which were used by the interviewer and observer to reflect the important topics addressed during the interview immediately after the interview. The video records were transcribed immediately afterwards by a research assistant or LK and corrected by the other. Transcripts were not returned to participants for comment and/or correction.

### Data analysis and integration of qualitative and quantitative findings

We used descriptive statistics to describe the samples for both the questionnaire and the interviews. We approached the analysis in two steps. In the first step we used descriptive statistics on the questions about the extent to which medical specialists provide CWIC. We proceeded with exploring the medical specialists’ perception of their professional role-responsibility in the second step. In this step we used both the questionnaire and the interviews to extract the specialists’ perceived role-responsibility in providing CWIC.

With regard to the analysis of the qualitative data, thematic analysis according to the guidelines of Braun and Clarke was applied [19]. We identified the themes and subthemes on a semantic level using a realist approach to show and summarize the patterns in the data. By using a realist approach we assumed that what a participant expressed was a straightforward reflection of their motivation and experience [29]. The transcripts were coded by LK, and identified themes were discussed with AdW until both agreed upon the correct interpretation of the findings. The final code tree was discussed with the whole research team.

During the iterative process of the qualitative analysis, we discovered that the medical specialists’ perception of their professional role-responsibility did not correspond with our previous understanding of CWIC based on the patients’ perspective [4]. The specialists recognized the four groups of patient work-concerns as presented to them during the interviews. However, when they described how they provided CWIC or expressed what they could or should do, their actions did not align to these groups. Actions taken by medical specialists revealed themselves to be guided by a process of clinical reasoning [30], in which the various patient concerns were addressed in a sequence that was logical for the physician. To continue with the analysis, we defined the professional role-responsibility as any action taken by the specialists described in their current practice (e.g., answering a letter from an occupational health physician) or any action they regarded a medical specialist could or should take.

The beliefs of each specialist regarding why they engaged in this practice, or why they believed they or other medical specialists could or should do so, were also coded. The coding scheme was inspired by the reasoned action approach [31, 32]. This approach suggests that the single best predictor of behavior is someone’s intention to perform that behavior. This intention is determined by the person’s behavioral beliefs, normative beliefs, and control beliefs, which form the person’s attitude, perceived norms, and perceived behavioral control. These beliefs were used to understand why some parts of CWIC were regarded to be the professional role-responsibility of the specialist and other parts were not.

**Fig. 1:**
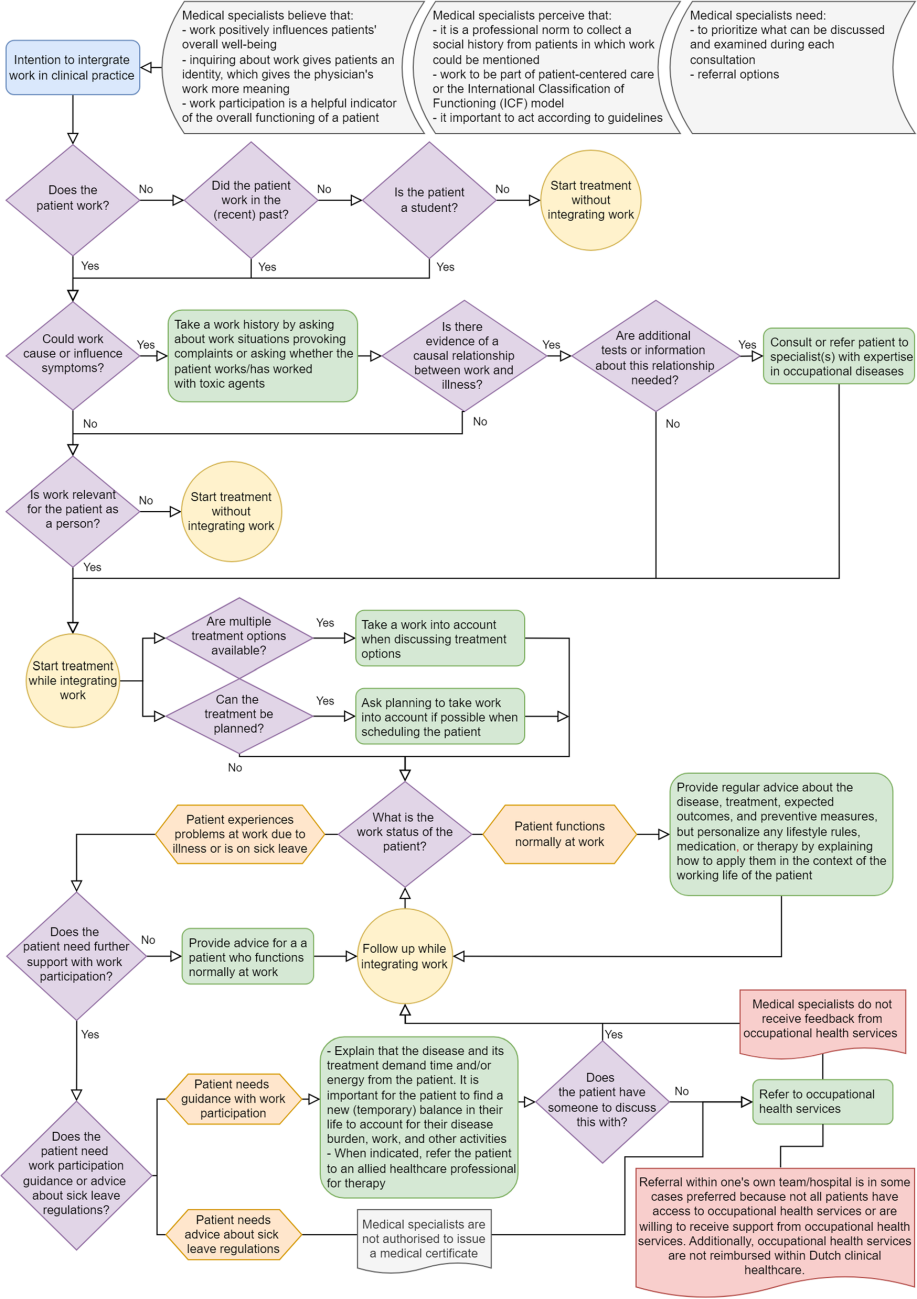
Clinical reasoning process leading to the different actions medical specialists considered they could take when intending to provide CWIC, and the main underlying beliefs, norms, and boundaries of the specialists. The clinical reasoning process begins with the assessment of the relevance of the topic of work during diagnostics. Subsequently, its relevance for the patient is determined. When treatment involves the integration of work, it may be considered when discussing treatment options (theme 1). Depending on whether the patient encountered any work-related problems or not, different advice is appropriate, but all advice predominantly focuses on health and disease management (theme 2). When the patient requires additional work-related support, a specialist will refer them to occupational health services. However, study participants noted that this is when they encountered the most limitations in providing CWIC due to barriers related to the Dutch healthcare system (theme 3)

Finally, to refine our understanding of providing CWIC, we structured the actions and the underlying beliefs leading to these actions in a summarizing flowchart (Fig. 1). To strengthen the findings from the interviews, we integrated the items on perceptions of the medical specialists’ professional role-responsibility from the questionnaire, which we analyzed with descriptive statistics. Participants did not provide feedback on the findings.

All qualitative analysis was conducted using MAXQDA 2020 [33]. SPSS version 28 was used for all statistical analyses [34].

### Research ethics

The general principles of research ethics outlined in the 2013 World Medical Association Declaration of Helsinki were applied [35]. Our study sample consisted of healthcare professionals questioned about their profession, and therefore ethical approval was not required as this is not subject to the Dutch law of medical scientific research (WMO) [36]. All participants volunteered. Informed consent was assumed when participants started the online questionnaire. All interviewees were provided with a participant information sheet prior to the interview and returned a written informed consent form. For confidentiality reasons, we cannot provide full details of the interview sample.

## Results

### Background characteristics

In total, 160 medical specialists started the questionnaire of which 129 completed it fully (response rate is unknown due to sampling method). The majority of the sample consisted of non-surgical specialists other than rehabilitation (71%), one fifth consisted of rehabilitation specialists (22%), and a small part consisted of surgical specialists (8%) (Table 1). Experienced medical specialists were represented more than less experienced specialists (56% > 10 years of experience versus 44% with < 10 years of experience). More female practitioners were represented than males (64% versus 36%). The specialists estimated that half of their patients worked. Finally, most specialists treated patients with chronic conditions as opposed to acute conditions.

**Table 1:** Background characteristics of the questionnaire and interview sample

Characteristics	Questionnaires		Interviews	
	Frequency	%^a^	Frequency	%^a^
Total	160	100	11	100
Completed full questionnaire	129	80.6	n/a	
Medical specialty				
*Non-surgical specialties other than rehabilitation*	*113*	*70.6*	*5*	*45.5*
Cardiology	2	1.3	1	9.1
Dermatology	75	46.9	1	9.1
Gastroenterology	7	4.4	1	9.1
Internal medicine	10	6.3	-	-
Neurology	7	4.4	-	-
Pulmonology	10	6.3	2	18.2
Rheumatology	2	1.3	-	-
*Rehabilitation specialties*	*35*	*21.9*	*5*	*45.5*
Rehabilitation medicine	31	19.4	5	45.5
Sports medicine	4	2.5	-	-
*Surgical specialties*	*12*	*7.5*	*1*	*9.1*
Cardio-thoracic surgery	1	0.6	-	-
Gynecology	1	0.6	-	-
Orthopedic surgery	7	4.4	1	9.1
Urology	3	1.9	-	-
Years of experience				
In medical specialist training	18	11.3	1	9.1
< 5 years	20	12.5	-	-
5–10 years	32	20.0	2	18.2
10–20 years	41	25.6	-	-
> 20 years	49	30.6	8	72.7
Gender				
Female	103	64.4	6	64.4
Male	57	35.6	5	35.6
Estimation of working patients				
0–20%	3	1.9	n/a	
20–40%	25	15.6	n/a	
40–60%	80	50.0	n/a	
60–80%	48	30.0	n/a	
80–100%	4	2.5	n/a	
Treats mostly patients with chronic conditions^b^	140	87.5	n/a	
Treats mostly patients with acute conditions^b^	36	22.5	n/a	
Has specific expertise in work and health	n/a		9	81.8

^a^Calculated as percentage of total study sample

^b^Asked as two separate questions, thus combinations are possible

### The extent to which medical specialists provide CWIC

On average medical specialists usually asked their patients whether they worked (M = 3.13, SD = 0.86), and the conversation about work was usually started by the specialist (M = 2.91, SD = 0.76) (Table 2). Sometimes the patients initiated a conversation about work (M = 2.02; SD = 0.70), but this was seldom the result of a request from an occupational health or insurance physician (M = 0.93, SD = 0.79; M = 0.73, SD = 0.76, respectively). Taking a work history was less common; on average specialists reported sometimes doing this but the spread was wide (M = 2.16, SD = 1.22).

**Table 2:** The extent to which medical specialist provide CWIC

How often was CWIC provided?^a^	Number of responses	Mean (SD)
How often do you ask whether your patient works?	160	3.13 (0.86)
How often do you take a work history?	160	2.16 (1.22)
How often do you discuss the topic ‘work' with your patients…		
- on your initiative?	160	2.91 (0.76)
- on the patient’s initiative?	160	2.02 (0.70)
- in request of an occupational health physician?	160	0.93 (0.79)
- in request of an insurance physician?	160	0.73 (0.76)
How often are the subjects below discussed during your contact with patients?		
- Influence of work on disease (both causal relation with disease or worsening of symptoms)	160	2.41 (0.84)
- Influence of disease on work ability	160	2.52 (0.89)
- Influence of treatment on work ability	160	2.14 (0.90)
- Questions about legal aspects regarding sick leave or work disability	160	1.48 (1.06)
In case of contact with an occupational health physician, how often was this…		
- on your initiative?	129	0.95 (0.95)
- on the occupational physician’s initiative?	129	2.75 (1.05)
- in request of your patient?	129	1.16 (0.92)
In case of contact with an insurance physician, how often was this…		
- on your initiative?	104	0.29 (0.59)
- on the insurance physician’s initiative?	104	2.88 (1.14)
- in request of your patient?	104	0.91 (0.95)
**What means of contact were used?**^**b**^	**Number of responses**	**Frequency (%)**
What ways of contact with an occupational health physician were used?		
- Never had contact	160	31 (19.4)
- Written	160	119 (74.4)
- By telephone	160	29 (18.1)
- Other ways	160	2 (1.2)
What ways of contact with an insurance physician were used?		
- Never has contact	160	56 (35.0)
- Written	160	102 (63.7)
- By telephone	160	6 (3.8)
- Other ways	160	-

*Abbreviations:* *SD* standard deviation

^a^ Scores on a five-point Likert scale from never (0), to rarely (1), to sometimes (2), to usually (3), to always (4)

^b^ Respondents could choose several options and therefore do not count up until total

Seeking contact with an occupational health or insurance physician was seldom or never initiated by a medical specialist (M = 0.95, SD = 0.95 and M = 0.29, SD = 0.59, respectively). More often, it was reported that an occupational health and insurance physician sought contact with a medical specialist (M = 2.75, SD = 1.05 and M = 2.88, SD = 1.14, respectively), usually in writing (74% and 64% of cases of contact with an occupational health and insurance physician, respectively). Patient requests for contact with occupational health or insurance physicians did not occur often (M = 1.16, SD 0.92 and M = 0.91, SD = 0.95, respectively). A third (35%) of the specialists reported never having contact with an insurance physician, and 19% reported never having contact with an occupational health physician. During patient consultation, specialists discussed influence of work on disease, influence of disease on work ability, and influence of treatment on work ability “sometimes” to “usually" (M = 2.41, SD = 0.84, M = 2.52, SD = 0.89, M = 2.14, SD = 0.90, respectively). Legal aspects related to sick leave for work disability were rarely discussed (M = 1.48, SD = 1.06).

### The perceptions of medical specialists about their professional role-responsibility in providing CWIC

In total, 11 medical specialists participated in interviews (Table 1). Of these, five were non-surgical specialists other than rehabilitation, five were rehabilitation physicians, and one was a surgical specialist. Most interviewees had more than 20 years of experience. Nine interviewees had specific interest in the field of work and health, ranging from recent practical experience to years of applying CWIC and performing research on this topic. The mean duration of the interviews was 37 min (SD = 4).

After completing the thematic analysis, three themes were identified: 1) understanding that work and health (problems) are inextricably linked, 2) supporting work participation within a specialist’s expertise, and 3) possibilities and limitations of the Dutch healthcare system. These themes each describe the main actions medical specialists perceived to be their professional role-responsibility, and the last theme delves deeper into the challenges they faced when wanting to do more with CWIC. Figure 1 provides a summary of the themes displaying how all actions are linked, as perceived by medical specialists. The supporting quotes from the interviews and the statistical analysis of the questionnaire can be found in Table 3.

**Table 3:** Supporting quotes from the interviews and results of the questionnaire elucidating the role-responsibility of the medical specialist

Supporting quotes from the qualitative analysis	Corresponding question from questionnaire^a^	Number of responses	Mean (SD)^b^
**Theme 1. Understanding that work and health (problems) are inextricably linked**			
*1.1 Asking about work and identifying its relevance for further discussion*			
Q1.1a “I once had a patient with a progressive muscle disease who was a laboratory technician and had to pipette all day, and yes, with limited hand function. That becomes a problem at some point. So, she developed shoulder complaints, and that's why she came to me. And only after further questioning did it become clear where that actually came from. Then you can together analyze, like, where could that be coming from? And how could you do the work differently?” (R8, F, rehabilitation physician)			
Q1.1b “So then, I think as a healthcare provider, you should, a bit, feel it out and see if it's relevant at that moment, whether you should look at it yourself.. bring it up. In the sense of 'does the work issue come into play, or would you like to return to work, or is it not relevant?'” (R4, M, orthopedic surgeon)			
*1.2 Investigating work factors during diagnostics*			
Q1.2 “Well, I think that the pulmonologist traditionally is trained to always inquire about occupational history. Because we simply know that many occupational diseases are directly related to work or that causal agents may be present there. I believe pulmonologists have been quite well-trained in this aspect from the beginning and also ask about it.” (R11, F, pulmonologist)	Investigating work factors as cause of illness during diagnosis or during (stagnation of) treatment	126	3.87 (0.90)
*1.3 Incorporating the patient's work life into discussion of treatment options*			
Q1.3 “Important is that every specialist, who sees a patient, should actually ask what you do in daily life. This not only concerns work but also leisure activities, sports, and the like. But I think it's good to have an understanding of the situation in which someone functions, both in terms of family and professionally. Because that does matter for the treatment you provide. You can technically perform a wonderful treatment, but if that means someone can never work again, it's a different story. While another treatment might increase their employment opportunities, you should take that into account in your considerations.” (R10, M, rehabilitation physician)	Considering the impact of treatment on work ability and discuss alternative options for treatment if possible	129	4.03 (0.74)
**Theme 2. Supporting work participation within a specialist’s expertise**			
*2.1 Supporting work participation while keeping the main focus on the patient’s health*			
Q2.1a “Look, an occupational physician, ideally, is more accessible to the employee than the medical specialist. They can much better monitor the treatment and the course of the condition, just like a general practitioner does.” (R1, M, dermatologist)	Providing advice when work can be resumed (for example after surgery or hospitalization)	126	3.24 (1.09)
Q2.1b “Look, I work with liver disease, so you don't see much on the outside about what's really going on with the patient. So, it often comes down to talking about balance, in terms of energy, what you spend and what you have left for other things. If someone can work and then comes home at 6 o'clock and has to go straight to bed, they're not doing something right. Then we need to look at that and ask, 'Can that be done differently? And is it discussable? And if so, with whom?” (R3, M, gastroenterologist)	Providing advice on maintaining a balance between illness and functioning at work	129	3.82 (1.00)
Q2.1c “Often in the later stages of rehabilitation, not in the first few weeks, but after a month or two, I also have conversations with patients like 'you are at this point now, you can walk 100 m, but for your job, you need to be able to walk at least one kilometer in a day. How could you build up to that? What can you do, for example, to resume a few hours of work or do modified work?'” (R10, M, rehabilitation physician)	Providing advice on functional limitations caused by illness	129	3.95 (0.99)
Q2.1d “If you get chest pain every time you have to lift a heavy beam [as for example a construction worker], it's obviously not very convenient. But if you feel it a little, and it goes away immediately, and you know it in advance, and you can handle it, then it's often not that bad. So, there's quite a bit to say about that.” (R9, F, cardiologist)			
Q2.1e “We can explain what happens if you continue working like that. Especially what happens if you keep working without protective equipment or proper ventilation. So, you can get a bit ahead of things. I also talk about retraining sometimes, especially with young people, like 18-year-old welders who have been out of work for months. So yes, it’s like a gradual scale, but I do provide advice up to that point, indeed.” (R2, F, pulmonologist)	Discuss with your patient about stopping work or changing jobs if you believe this is better for your patient's health	129	3.36 (1.02)
*2.2 Paying attention to prevention of sick leave*			
Q2.2 “I also see quite a few patients again after 2 years. They have built up their routines nicely, working for 4 days, and they've been doing it for a year. Then they think, 'Oh, it's not that bad; I can go back to working from Monday to Thursday.' Or their supervisor thinks, 'It's fine; can't you just work that extra hour on the weekend or do some overtime?' And they start doing that, and then, they completely break down. Everyone thinks they have a burnout, but they don't; they just forgot that they have that brain injury. I see that group quite often. And now, we've tried to be proactive about it. So basically, people with brain injuries who stop rehabilitation and day treatment with us now receive an explanation about relapse, like, 'Even if you think things are going well, we have still demonstrated that those problems exist, so don't push yourself to do more.” (R5, F, rehabilitation physician)	Providing advice on preventive measures at work to prevent illness/worsening of symptoms/exacerbations	129	3.93 (0.87)
*2.3 Informing patients about the existence of regulations and where to find them*			
Q2.3 “The question is whether it belongs to the hospital? It is, of course, a very social aspect, about law and regulations […] [The responsibility to inform patients about this] actually begins earlier in the sense of, as far as we [as medical specialists] know something about it, when you say 'the, uh, sick leave, reintegration plan, is it made? who looks at it? are you aware that one year is a crucial moment in the bigger picture. Has there been any contact with you about it?' People generally know very little, I think. But that is a bit more the company's responsibility to keep in touch with its employees about it.” (R3, M, gastroenterologist)	Providing an information folder to the patient about laws and regulations regarding absenteeism or incapacity for work due to illness	119	2.70 (1.08)
**Theme 3. Possibilities and limitations within the Dutch healthcare system**			
*3.1 (Im)possibility of designating work participation as a treatment goal*			
Q3.1a “Yes, when [a work-related issue] comes up, when it's noticed [by a medical specialist], it shouldn't be left unaddressed, even if you don't have the time or knowledge for it yourself, at least they should refer it to someone else. Yes, I would really find it a waste, ultimately for the entire healthcare system, if it's not given attention. Because sick leave is, of course, a significant cost factor, and if you can prevent it, well, you can do a lot of analyses for that, so to speak, beforehand. I've just understood it's a different budget.” (R2, F, pulmonologist)	Collaborating with other professionals within the clinical health sector (such as social work, physiotherapist, occupational therapist, psychologist) to contribute to a solution for work-related problems	125	3.86 (1.02)
Q3.1b “So, I think that [work] is definitely an important question, but well, it's just very difficult because there's all these things that need to be discussed in such a consultation, in those 10 min. It's almost impossible.” (R9, F, cardiologist)			
Q3.1c "Then I think that the occupational health physician should be financed differently. A occupational physician is currently financed by the employer. […] What you actually need is an occupational health physician who works entirely in the clinic, who understands the profession of an occupational physician, where you as a medical specialist can refer the patient to investigate whether work plays a role in this illness. They can then start with an occupational physician and an occupational hygienist and set everything in motion. But that specialization does not exist." (R11, F, pulmonologist)			
Q3.1d “We also have the option to conduct a work assessment, both physical and mental work assessments. These are services that are not covered by health insurance. So, if you choose to have them done, the employer, UWV [i.e., Employee Insurance Agency], or personal injury compensation should cover the costs.” (R10, M, rehabilitation physician)			
*3.2 Cooperating with an occupational health physician*			
Q3.2a "When people are at home, the exposure is naturally gone; so then the problem is solved. But the biggest one is not, because the best thing is, of course, that people go back to work. But for health, we often undergo a rehabilitation process first. We assess whether we can function well again in a home situation because that has often become a problem. And then, of course, you have to see if they can go back to work, and that is again in consultation with the company doctor." (R2, F, pulmonologist)	Collaborating with occupational health physicians on issues related to work and health (so-called occupational-clinical health cooperation)	124	3.62 (0.87)
Q3.2b “When I simply look at the process, it is indeed quite a lot of information letters that come in. So, whether it's the company physician, an insurance physician, or in any case, someone writes a letter with a request for information, and you try to answer that politely, but there is little consultation beyond that.” (R4, M, orthopedic surgeon)	Providing information to the occupational health physician or insurance physician (UWV)	122	4.05 (0.74)
Q3.2c “These patients who come here for outpatient rehabilitation, we know them very well, with all the ins and outs. And you just can't put that on paper, so if, for example, you're concerned about a home situation. Yes, are you going to put that in such a letter, will that help the patient, you know? In a conversation, you can feel or notice much better what you can adjust a little better because it's often a bit of a give and take in these kinds of things, returning to work” (R5, F, rehabilitation physician)			
Q3.2d “But if I think about the conversation I had very recently, I believe it was yesterday, with an occupational health physician, well, during the phone call, it wasn't entirely clear to me what the interests of the occupational health physician are. They first mentioned that 'uh, yes, the company has implemented a reorganization, so his old positions are no longer available.' And I think, 'well, that's not an issue, that's your problem.' Because this gentleman has limitations in his functioning, I was asked 'to what extent is he really limited? What can't he do?' Well, in this case, I did indicate what he can't do because I believe I have medical grounds for it. But I can't foresee the exact consequences it will have for his future. So, I'm a bit cautious about it” (R7, M, rehabilitation physician)			

*Abbreviations: SD* standard deviation, *UWV* Employee Insurance Agency

^a^ The overall question in the questionnaire was: To what extent do you agree that the actions below are your responsibility?

^b^ Scores on a five-point Likert scale from strongly disagree (1), to disagree (2), to neutral (3), to agree (4), to strongly agree (5)

## Theme 1: Understanding that work and health (problems) are inextricably linked

### Asking about work and identifying its relevance for further discussion

When a medical specialist has the intention to provide CWIC, the clinical reasoning process starts with asking a patient about his or her occupation and exploring whether it is relevant to further discuss this topic. Medical specialists perceived this to be part of patient-centered care and fitting within the ideas of the International Classification of Functioning, Disability, and Health (ICF) [37]. They saw that work participation was an important topic for many of their patients and believed that being able to work would improve the patient’s overall well-being and, as such, could contribute to the patient’s health. Discussing work with patients could be relevant when work might be a factor causing the disease (Q1.1a) or when the disease caused problems for work participation (Q1.1b). Unraveling this interaction between work and disease enabled the medical specialist to comprehend their patient as a unique individual, rather than merely as a clinical case to be managed. This recognition of the patient’s broader identity, shaped by contextual factors such as work, enhanced the physician's sense of purpose and made their role more meaningful.

### Investigating work factors during diagnostics

For some diseases, it is necessary to investigate whether work factors caused or influenced the patient’s symptoms to conclude diagnostics (Q1.2), which was reflected in the questionnaire findings (M = 3.87, SD = 0.90). Diseases to which this applies are those where such investigations impact the treatment plan (e.g., an injury caused by a work accident does not need further investigation than acknowledging the fact that this was the cause of injury, but a repeated strain injury due to work activities does need attention). Detecting a causal relation between work and symptoms can entail taking a work history by asking about work situations that are provoking the complaints, asking whether the patient works with harmful agents (or did so in the past), or consulting specialists of occupational diseases.

### Incorporating the patient's work life into discussion of treatment options

The medical specialists also believed that participation in work could influence the outcome of the treatment (Q1.3). To support work participation during treatment, the specialists described incorporating the context of a patient's working life into discussions of treatment options and planning when possible. On average the questionnaire respondents agreed it was important to consider the impact of treatment on work ability into discussions of treatment options (M = 4.03, SD = 0.74).

## Theme 2: Supporting work participation within a specialist’s expertise

### Supporting work participation while keeping the main focus on the patient’s health

Medical specialists believed that their patient’s work status is a useful indicator of the overall functioning during treatment. However, the practitioners’ main priority is to support the patient in maintaining or returning to good health. This was reflected by the neutral responses they gave in the questionnaire about providing advice as to when work can be resumed (M = 3.24, SD = 1.09). The interviewed specialists explained that they did not have the knowledge or expertise to provide meaningful advice about returning to work, and they regarded occupational health physicians as the experts in this domain (Q2.1a).

Instead, medical specialists who believe that a patient’s work status is an insightful indicator of overall functioning differentiates between patients with and without problems at work due to illness. For patients without work problems, specialists reported providing regular advice about disease, treatment, expected outcomes, and preventive measure, but striving to support patients in integrating this advice into the context of the patient’s working life. For patients with work limitations, a specialist will orient their advice towards keeping a healthy balance between disease burden, other activities, and work participation, and prompt the patient to discuss this with someone with knowledge of the patient’s work such as their occupational health physician (Q2.1b).

Depending on the specific medical condition of the patient, the details of practitioners’ advice about achieving a sustainable work-illness balance will differ. For patients in the sub-acute period after an event, this advice could be to limit certain activities temporarily (e.g., avoid heavy lifting in the first six weeks after surgery)—as agreed by the questionnaire respondents (M = 3.95, SD = 0.99). In case of a more severe injury, they will discuss with the patient what therapy is needed to return to certain activities at work (Q2.1c). Specialists treating patients with chronic diseases perceived it to be their role to advise patients in finding balance between the energy needed to cope with the disease burden, daily activities at home, and work—as reflected by the questionnaire answers (M = 3.82, SD = 1.00)—or in coping with complaints during work (Q2.1d). Finally, the medical specialists recognized that some (progressive) diseases make it inevitable that a patient must reduce their work activities. To maintain a healthy balance for this group, a few specialists regarded it to be their role to advise that patients consider more drastically reducing or changing work (always leaving the final decision up to the patient or the occupational health physician)—which is reflected by the more neutral responses in the questionnaire (M = 3.36, SD = 1.02) (Q2.1e).

### Paying attention to prevention of sick leave

As a part of providing regular advice, several specialists indicated the importance of (secondary) prevention to ensure that a patient avoids (recurrent) sick leave due to a (chronic) condition. They reasoned this would reduce the strain on the use of specialized medical care. Thus, they perceived it to be their role to employ medical interventions within their expertise to prevent sick leave (Q2.2). This was also reflected by the questionnaire findings (M = 3.93, SD = 0.87).

### Informing patients about the existence of regulations and where to find them

With the exception of the rehabilitation physicians, medical specialists in this study perceived addressing questions about regulations related to disease and work to *not* be within their role-responsibility, corresponding with the questionnaire findings (M = 2.70, SD = 1.08). In comparison with the other medical specialists, rehabilitation physicians often had the possibility and time of explaining these regulations (e.g., by sending patients to the social worker within their team) because not offering this information could interfere with a patient’s rehabilitation progress. All other medical specialists perceived it within their role to provide support by reminding the patient of his or her own legal responsibilities to seek information and/or by directing the patient to where to find this information (e.g., from their employer or the Employee Insurance Agency (in Dutch: UWV)) (Q2.3).

## Theme 3: Possibilities and limitations within the Dutch healthcare system

### (Im)possibility of designating work participation as a treatment goal

All interviewed specialists emphasized that they increasingly regarded enhancing work participation as a possible treatment goal. Rehabilitation physicians in particular mentioned that they perceive this as a professional norm since medical guidelines covering the topic of work were introduced. However, the consensus was that medical specialists can only signal a work participation problem and should refer patients to the appropriate professional when more guidance is needed (M = 3.86, SD = 1.02) (Q3.1a), because specialists need to prioritize what topics they focus on during each consultation (Q3.1b).

To provide guidance on work-related issues for patients, medical specialists mentioned referring patients to nurse specialists or social workers within their clinic or to any allied healthcare professional outside their clinic. However, referral to occupational physicians was also deemed essential. Nevertheless, specialists reported that referring patients to occupational health services as often problematic. In many cases contact with the patient’s occupational health physician could not be established.

The reasons for this lack of contact the specialists mentioned were that many patients do not know who their occupational health physician is, some patients do not have access to an occupational health physician, and other patients do not want the specialist to contact their occupational health physician. Even when the occupational health physician is known, it can be difficult for specialists to obtain the occupational physician’s contact information or to establish communication with them due to conflicting time schedules. Therefore, some specialists advocate for a less strict separation between the occupational and clinical domains to make occupational health physicians more accessible. They believe this approach could ultimately benefit patient well-being (Q3.1c). To integrate occupational health services into the clinical domain, medical specialists indicated several initiatives to provide occupational health services within the hospital (e.g., by enlisting a clinical occupational consultant). Still, this was not always feasible, they explained, because this care is not reimbursed within the clinical healthcare system in the Netherlands (Q3.1d) (which resulted in the discontinuation of these initiatives in some clinics).

### Cooperating with an occupational health physician

Medical specialists recognized the added value of cooperating with occupational health physicians for two main reasons. First, they acknowledged that occupational health physicians’ specific expertise and knowledge about a patient's workplace (e.g., investigating workplace-related factors in cases like allergic contact dermatitis) could have added value for refining the treatment plan. Reciprocally, specialists could also lend their specific knowledge of rare diseases to occupational health physicians to aid in a patient’s reintegration plan (Q3.2a). Second, the specialists explained that occupational health physicians assist patients in navigating the obligatory steps set out by the Gatekeeper Improvement Act (i.e., the Dutch sick leave procedure lasting up to two years after initial sickness absence). Indeed, the specialists reported that they occasionally perceived it to be their role to support the patient in expediting this process, which may involve contacting an occupational health physician. However, intensive contact is typically considered unnecessary, and respondents emphasized patient responsibility in accomplishing work participation as treatment goal. As such, there was not a broad consensus that specialists should cooperate with the occupational health physician in the questionnaire responses (M = 3.62, SD = 0.87).

According to specialists, patients are responsible for maintaining contact with their occupational health physician, which is within the boundaries of current European privacy legislation. Within the relationship between patient, medical specialist, and occupational health physician, the communication flow between the medical professionals primarily consisted of a one-way communication flow. In this communication flow specialists primarily respond to the occupational health physician's letters requesting medical information (Q3.2b). None of the interviewed specialists had ever experienced receiving a report back from the occupational health provider about what happened with this information. Although most specialists agree that it is their role-responsibility to provide the requested information (M = 4.05, SD = 0.74), the specialists regarded this situation as *not* ideal in all cases and reported sometimes preferring verbal contact, such as phone or video call. Verbal contact would allow them to convey nuances in the patient's medical situation that are challenging to express in written correspondence (Q3.2c). However, specialists also mentioned that they approached verbal contact cautiously due to past negative experiences and uncertainties regarding occupational health physicians’ priorities, given the Dutch legal structure where this physician stands between the patient/employee and their employer (Q3.2d).

## Discussion

This mixed-methods study aimed to assess the extent to which medical specialists provide CWIC and, secondly, to gain insight into medical specialists’ perceptions of their professional role-responsibility with relation to CWIC. While specialists reported that they often asked whether their patients work, they reported only sometimes delving deeper into the topic by taking a work history. Specialists employed clinical reasoning to integrate work-related questions into diagnostics and treatment plans within their medical expertise. Within these plans, specialists prioritized patient health while supporting work participation. Their advice revolved around maintaining a balance between disease, work, and other activities, with the ultimate goal of helping the patient maintain or return to good health. Furthermore, specialists encountered limitations within the healthcare system in supporting patients with work-related issues.

In our survey, medical specialists indicated that they often asked about their patients' work, usually on the specialist’s initiative, but only occasionally delved deeper into the topic by taking a work history. This aligns with findings in the literature, where medical specialists, to varying degrees, have been shown to inquire about work but not to consistently integrate the topic into their diagnostics or treatment plans [38–40]. The interviewed specialists in our study emphasized that for the topic of work to be integrated into diagnostics or treatment plans, it must be either medically relevant or pertinent to the patient's well-being. To assess this relevance, specialists employ clinical reasoning. Furthermore, the specialists in our study explained that they consider the topic of work to be important for discussion. Independent of their specialty, they believed that work participation positively influenced their patients' overall wellbeing and that inquiring about work gave their patients an identity which, in turn, gave their work as physicians more meaning. Finally, work participation was believed to be a helpful indicator of the overall functioning of the patient. This could elucidate why medical specialists in our study indicated taking initiative to discuss work, in contrast to other studies wherein discussions about work were reported to be initiated by the patients themselves [41–43].

In the interviews in our study, the medical specialists offered two reasons for why they regarded discussing work to be an important aspect of patient consultation to consider. First, asking about work was considered important within the framework of patient-centered care [44, 45]. Patient-centered care principles (such as considering the patient holistically or involving patients in shared decision-making) are increasingly being integrated into clinical healthcare [46–49]. This framework explains why specialists may not explore the topic of work further; when patients themselves do not attach significance to work and it exerts no discernible influence on their specific medical condition (e.g., no exacerbation of symptoms due to work), most specialists see no added value in probing further. The second reason work was considered important that emerged from the interviews was the advent of guidelines addressing the topic of work, which specialists consider instrumental in shaping their professional standards. The rehabilitation physicians referred to such guidelines and considered it inadequate care not to address the topic of work, while other specialists noted that their guidelines often do not accommodate the topic of work and discussed their personal incentives why they regarded work an important topic. Multiple other studies have noted the absence of guidelines for providing work-related support as well [42, 50–57]. This highlights the need for a structural embedment of the topic of work within medical guidelines to stimulate medical specialists to provide CWIC.

However, the interviewed specialists mentioned that after initially asking about work, they perceived it to be their role-responsibility to provide disease-specific advice that could influence work participation. Furthermore, they considered it their role to recognize and refer patients when work-related issues become apparent. They experienced time constraints during patient consultations and acknowledged the expertise of occupational health physicians. Seeking support from other professionals for work-related problems has been mentioned by medical specialists in many other studies as well [51, 52, 56, 58–64]. Similarly to other studies, cooperation with occupational health professionals was reported in our study to be challenging for medical specialists [65, 66]. In the Netherlands, occupational healthcare services are not reimbursed through regular private healthcare insurers but are financed direct by employers [67–69]. This contributes to the limited provision of occupational health services in clinical healthcare as specialists in our study described. Although untrue [70], this financing structure creates the appearance for some patients that the occupational health physicians’ interests align more with the employer than with the patient/employee [4, 10]. Such suspicions enhanced some specialists’ hesitancy in actively contacting occupational health physicians. Additionally, patients sometimes find it difficult to navigate connecting to their occupation health physician through the employer [11]. This makes it challenging for medical specialists to directly contact the patient’s occupational health physician, as our sample of specialists reported. Despite these barriers, specialists believed that discussing work with the patient should be part of treatment, and they might refer patients to other appropriate resources, such as nurses, or social workers, and encourage the patient to contact their occupational health physician.

### Methodological considerations

Our mixed-methods study design effectively allowed us to integrate quantitative and qualitative data to gain better insight into Dutch clinical practices. Patient expectations regarding discussing work-related issues with medical specialists from our previous research [4] were systematically surveyed among a substantial group of medical specialists. This revealed that the specialists recognized the patients work-related issues, except in the area of explaining sick leave regulations. By conducting interviews following the questionnaire, we gained deeper explanations from the medical specialists [17].

Due to the unavailability of a validated questionnaire for measuring the extent of CWIC provision and specialists’ perception of role-responsibility, we created a customized questionnaire. To ensure the questionnaire's acceptability, we conducted a pilot to assess its face validity. While our study was not designed to evaluate the content validity of the questionnaire, employing a mixed-methods study design involving sequential interviews following the questionnaire provided valuable insights into its content validity [71]. However, for further research on the extent of CWIC provision, developing a validated questionnaire might be considered.

We acknowledge potential selection bias in both the interview and questionnaire samples. The questionnaire sample was susceptible to selection bias, with a notable presence of dermatologists. Given the voluntary recruitment approach, those who completed the questionnaire likely had a strong interest in the subject. In our interview sample, it was evident that specialists had a positive attitude toward CWIC. Indeed, for rehabilitation physicians it had become their professional standard to address work participation, so this subgroup might be a good representation of rehabilitation practice. However, addressing work being the professional standard does not account for the other specializations and could have resulted in a selection bias. This possibly overestimates the extent to which specialists provide CWIC.

Our recruitment approach possibly also caused a difference between the survey sample and the interview sample (i.e., a specialist with an interest in the topic might be more likely completed the questionnaire, a specialist with a *special* interest, in turn, might be more inclined to subsequently enlist for an interview). In the terms of Rogers et al. [72] from the field of implementation research, the interviewed specialists may be considered to be innovators and early adopters, whereas the questionnaire sample likely represented the (early) majority, who do not yet provide CWIC. The laggards are not represented by our sample. This differentiation is all valuable information: the innovators and early adopters shed light on the challenges faced in adopting a positive attitude towards providing CWIC, while the questionnaire responses reveal current practice (where CWIC is of only minor importance).

### Implications for practice and research

This study illuminates what patients or other healthcare providers could reasonably expect from medical specialists in delivering CWIC. Specialists considered it within their capacity to offer advice in balancing disease burden and work participation or other forms of societal participation. Such advice may include acknowledging that during a post-medical event physical recovery phase, patients may temporarily experience activity limitations, prompting a need to find balance between these limitations in daily activities *and* their work. While a specialist can clarify these limitations, it is ultimately the patient who must discover a balance within their life. Advice may also entail adjusting treatment plans through collaboration with patients (i.e., adjusting medication for workdays to manage symptoms) provided it is medically justifiable.

Medical specialists' willingness to discuss work within the context of person-centered care is commendable. However, they wish to stay within their own expertise, focusing on improving the patient's health, flagging work-related problems, and referring patients to occupational health care professionals when necessary since they cannot offer vocational guidance themselves.

In many healthcare systems, cooperation between clinical and occupational healthcare is challenging due to financial constraints and other barriers [68, 73, 74]. This was the case for the medical specialists in our sample. To bridge this gap and facilitate closer cooperation, professionals from various domains, such as medical specialists, occupational health physicians, and healthcare policymakers, could establish formal channels for communication and information sharing [74, 75]. This might include creating protocols for patient referrals, creating multidisciplinary team opportunities for joint care planning and case-conferencing, facilitating joint training programs, and improving financial reimbursement for CWIC. Such efforts could foster a more coordinated approach to healthcare, ensuring that patients receive comprehensive support both for their health and their work-related needs.

Interestingly, our interviewees explained that the topic of work became more significant for them as they gained experience over the years. This suggests that experience plays a role in recognizing the importance of work in healthcare and underscores the potential need for increased emphasis on patient needs during medical training. Additionally, general education and training in work and health might endorse this. Further research could center on reinforcing the recognition and emphasis of the importance of work for patients and on the relation between work and health among medical specialists in order to promote the integration of work as a topic in their daily practice. Subsequently, research should concentrate on enhancing the capacity of medical specialists to provide CWIC, as demonstrated by recent studies [76, 77].

## Conclusion

While most specialists in our study asked whether their patients worked, they often did not explore the patients’ work history. Specialists perceived that inquiries about work could be part of their professional role-responsibly, but they prioritized patient health while endorsing work participation. Specialists integrated work-related questions into diagnostics and treatment plans and focused their advice on achieving a balance between disease burden, other activities, and work participation. For other work-related support needs, specialists prefer to refer patients to appropriate providers. However, limitations within the Dutch healthcare system hinder the provision of comprehensive work-integrating support. This highlights the need for improved strategies within the healthcare system to address and support patients with work-related concerns.

## Supplementary Information

Supplementary Material 1.

Supplementary Material 2.

## Data Availability

The datasets generated and analysed during the current study are not publicly available due to individual privacy could be compromised but are available from the corresponding author on reasonable request.
