# Supplementary material for: The extent to which medical specialists provide Clinical Work-Integrating Care (CWIC) and their perceived role-responsibility: a mixed-methods study
Source: BMC Health Serv Res. 2025 Mar 27;25:448. doi: 10.1186/s12913-024-12137-y (PMC11948937; doi:10.1186/s12913-024-12137-y)
Supplement: Supplementary file 1 — Supplementary Material 1. [file 12913_2024_12137_MOESM1_ESM.docx]

**Survey ‘Attention to work in the consultation room’**

[Introduction to survey]

Health is increasingly perceived as the ability to cope with life’s challenges.^1,2^ One of these challenges is work.^3^ As a result, the patient, society, and government more often ask the medical specialist to, in one way or another, deal with the factor work.^4^ This is called *clinical work-integrating care.* The healthcare professional acts on the notion that work and health are related and addresses work within the clinical context.

The aim of this survey is to determine *your* opinion as a medical specialist about the content and form of clinical work-integrating care, as well as what part you can play in it. An inventory is also made of the extent to which clinical work-integrating care is already provided in current practice.

To complete this survey, it is not necessary that you currently provide and form of clinical work-integrating care yourself.

Duration: 10 min

^1^ Federatie Medisch Specialisten. Visiedocument Medisch Specialist 2025. Ambitie, vertrouwen, samenwerken Utrecht: FMF. 2017.
^2^ Huber M, Knottnerus JA, Green L, Horst Hvd, Jadad AR, Kromhout D, et al. How should we define health? BMJ. 2011;343:d4163 ^3^ KNMG. KNMG-visiedocument Zorg die werkt: Naar een betere arbeidsgerichte medische zorg voor (potentieel) werkenden. Utrecht: KNMG; 2017. ^4^ Brief van voormalig minister Koolmees, 2020/21, 25883, nr. 416

| Variabele name | Question | | Answer options |
| --- | --- | --- | --- |
| *Demographics* | | | |
| MedSpecialist | Are you a medical specialist or in training to become a medical specialist? | | Yes, medical specialists / Yes, in training to become a medical specialist / Yes, but not practicing / No [ if ‘Yes, but not practicing’ or ‘No’, end of survey] |
| Specialisme | What is your specialism? | | List of specialisms [drop down menu] |
| SpecialismeAnders |  | [If ‘other’ to the above question]  What is your specialism if it is not listed? | [Short free text] |
| WerkErvaring | How many years of experience do you have as a medical specialist?  [i] This is excluding your years in training to become a medical specialist  [Note: only for medical specialists, not for those in training] | | <5 years / 5-10 years/10-20 years/> 20 years |
| Geslacht | What is your gender? | | Male / Female |
| PercWerkendePtn | What do you estimate is the percentage of your patient population that works? | | 0-20% / 20-40% / 40-60% / 60-80% / 80-100% / I don’t know |
| TypePtChron | Do you mainly see patients with chronic conditions? | | Yes / No |
| TypePtAcuut | Do you mainly see patients with acute conditions? | | Yes / No |
|  |  | | |
| *Questions about discussing work in current practice* | | | |
| [Objective: to determine the extent to which attention is paid to work in current practice] | | | |
| VragenNaarWerk | Can you please indicate how often this occurs in the questions below?   1. How often do you ask if your patient is working? 2. How often do you take a work history [i]?   [i] A work history includes questions such as: ‘Are you currently employed?’ ‘What kind of work do you do?’ ‘Are you currently out of work?’ ‘And which health complaints play a role in this?’ ‘Do you work in shifts?’ ‘Are your health complaints lessened when you don’t work, for example, at the weekend or during the holidays?’ ‘Do you have work problems?’ ‘Are you in contact with an occupational physician?’ | | Always/usually/sometimes/rarely/never  Always/usually/sometimes/rarely/never |
| InhoudWerkVragen | How often are the topics below discussed during your contact with patients?   1. Influence of work on illness (as a cause of illness or worsening of symptoms) 2. Influence of illness on work ability 3. Influence of treatment on work ability 4. Questions about legislation and regulations regarding absenteeism from work or disability due to illness   [i] This can concern both the influence of work factors on illness and the influence of illness on work participation. | | Always/usually/sometimes/rarely/never  Always/usually/sometimes/rarely/never  Always/usually/sometimes/rarely/never  Always/usually/sometimes/rarely/never |
| WiensInitiafiefWerk | On whose initiative do you discuss the topic of ‘work’ with your patients?   1. On my own initiative 2. On the patient’s initiative 3. At the request of the occupational physician 4. At the request of the insurance physician of the social security agency | | Always/usually/sometimes/rarely/never  Always/usually/sometimes/rarely/never  Always/usually/sometimes/rarely/never  Always/usually/sometimes/rarely/never |
| ContactBA | Do you ever have contact with an occupational physician? | | No  Yes, in writing  Yes, by phone  Otherwise  [multiple answer options possible] |
| ContactBAtoelichting |  | [if ‘other’ to the above question]  Can you explain this? | [Short free text] |
| ContactBAwie |  | [If no ‘no’ to the above question]  On whose initiative did this contact come about?   1. I contacted the occupational physician 2. The occupational physician contacted me 3. My patient asked me to make contact | Always/usually/sometimes/rarely/never  Always/usually/sometimes/rarely/never  Always/usually/sometimes/rarely/never |
| ContactVA | Have you ever had contact with an insurance physician (social security agency)? | | No  Yes, in writing  Yes, by phone  Yes, in other ways, namely …  [multiple answer options possible] |
| ContactVAtoelichting |  | [if ‘other’ to the above question]  Can you explain this? | [Short free text] |
| ContactVAwie |  | [if no ‘no’to the above question]  On whose initiative did this contact come about?   1. I contacted the insurance physician 2. The insurance physician contacted me 3. My patient asked me to make contact | Always/usually/sometimes/rarely/never  Always/usually/sometimes/rarely/never  Always/usually/sometimes/rarely/never |
|  |  | | |
| *Statements about clinical work-integrating care (attitude, self-efficacy, and social norm)* | | | |
| [Objective: to measure attitude, self-efficacy, social norms, and other factors that play a role in clinical work-integrating care]  *Explanatory note: statements are based on personal determinants already found from the problem model* | | | |
| [part ‘attitude’] | | | |
| AGMZAttitude | To what extent do you agree with the following statements? | | |
|  | 1. I think it is important to discuss work with my patients | | Strongly agree / agree / neutral / disagree / strongly disagree |
|  | 1. I believe that I should pay attention to work during the treatment process | | Strongly agree / agree / neutral / disagree / strongly disagree |
|  | 1. I think it is important to view patients as a whole, work is part of that | | Strongly agree / agree / neutral / disagree / strongly disagree |
|  | 1. I think work is an important outcome measure in treatment | | Strongly agree / agree / neutral / disagree / strongly disagree |
|  | 1. In order to practice my specialty, asking about the work history can be a valuable addition to providing quality care (e.g., a correct diagnosis) | | Strongly agree / agree / neutral / disagree / strongly disagree |
|  | 1. If my patient’s goal is to be able to work, I think it is important to take this into account in the treatment plan. | | Strongly agree / agree / neutral / disagree / strongly disagree |
|  | 1. I believe that giving advice about work is not the task of the medical specialist | | Strongly agree / agree / neutral / disagree / strongly disagree |
|  | 1. If I receive a question from the patient about his or her work, I am not allowed to make any statements about it | | Strongly agree / agree / neutral / disagree / strongly disagree |
| [part ‘social norm’] | | | |
| AGMZSocialNorm | To what extent do you agree with the following statements?  [i] ‘My colleagues’ refers to medical specialists with whom you work and who treat a similar patient population as yourself | | |
|  | 1. My colleagues find discussing work with patients important | | Strongly agree / agree / neutral / disagree / strongly disagree |
|  | 1. My colleagues pay attention to work participation during the treatment process | | Strongly agree / agree / neutral / disagree / strongly disagree |
|  | 1. My colleagues think it is important to views their patients as a whole, work is part of that | | Strongly agree / agree / neutral / disagree / strongly disagree |
|  | 1. My colleagues consider work an important outcome measure in successful treatment | | Strongly agree / agree / neutral / disagree / strongly disagree |
|  | 1. Within my specialty, my colleagues consider taking a work history important for providing quality care (e.g., a correct diagnosis) | | Strongly agree / agree / neutral / disagree / strongly disagree |
|  | 1. If a patient’s goal is to be able to work, my colleagues think it is important to take this into account in the treatment plan. | | Strongly agree / agree / neutral / disagree / strongly disagree |
|  | 1. My colleagues believe that giving advice about work is not a task for medical specialists | | Strongly agree / agree / neutral / disagree / strongly disagree |
|  | 1. My colleagues regularly consult with their patient’s occupational physician | | Strongly agree / agree / neutral / disagree / strongly disagree |
| [part self-efficacy, knowledge and other factors] | | | |
| AGMZSelfEfficayOther | To what extent do you agree with the following statements? | | |
|  | 1. If I receive a question from my patient about his or her work, I have sufficient knowledge to advise on this | | Strongly agree / agree / neutral / disagree / strongly disagree |
|  | 1. I have sufficient information at my disposal (such as guidelines) to answer work-related questions | | Strongly agree / agree / neutral / disagree / strongly disagree |
|  | 1. I know what tasks an occupational physician performs | | Strongly agree / agree / neutral / disagree / strongly disagree |
|  | 1. I know what tasks an insurance physician (from the social security agency) performs | | Strongly agree / agree / neutral / disagree / strongly disagree |
|  | 1. I don’t have time to deal with work-related questions from my patients | | Strongly agree / agree / neutral / disagree / strongly disagree |
|  | 1. I fear legal consequences when giving advice about work | | Strongly agree / agree / neutral / disagree / strongly disagree |
|  | 1. I will not contact an occupational physician or insurance physician due to privacy legislation | | Strongly agree / agree / neutral / disagree / strongly disagree |
|  | 1. I do not answer questions from the occupational physician, because I do not receive financial compensation for this | | Strongly agree / agree / neutral / disagree / strongly disagree |
|  | 1. I do not answer questions from the occupational physician, because I never receive feedback about what is done with the data | | Strongly agree / agree / neutral / disagree / strongly disagree |
|  |  | | |
| *Questions about the interpretation of one’s tasks and responsibilities (Attitude)* | | | |
| [Objective: measure attitude about the interpretation of one’s tasks and responsibilities of specific components within clinical work-integrated care]  *Explanatory note: items are based on a model constructed following research with patients* | | | |
| Taakopvatting | Below are a number of actions related to ‘work’ which patients mentioned during a previous study.  To what extent do you agree that these actions are your responsibility? | | |
|  | 1. Investigate work factors as causes of disease during diagnosis or during (stagnation of) treatment | | Completely agree/agree/neutral/disagree/completely disagree/no opinion/not applicable |
|  | 1. Take into account the influence of treatment on work ability and discuss alternative options for treatment if possible | | Completely agree/agree/neutral/disagree/completely disagree/no opinion/not applicable |
|  | 1. Provide advice on maintaining a balance between illness and work performance | | Completely agree/agree/neutral/disagree/completely disagree/no opinion/not applicable |
|  | 1. Provide advice on when work can be resumed (for example, after a procedure or hospital admission) | | Completely agree/agree/neutral/disagree/completely disagree/no opinion/not applicable |
|  | 1. Provide advice on functional limitations caused by illness | | Completely agree/agree/neutral/disagree/completely disagree/no opinion/not applicable |
|  | 1. Provide advice on preventive measures at work to prevent illness/worsening of symptoms/exacerbations | | Completely agree/agree/neutral/disagree/completely disagree/no opinion/not applicable |
|  | 1. Talk to your patient about stopping work or changing jobs if you believe this will benefit the patient’s health | | Completely agree/agree/neutral/disagree/completely disagree/no opinion/not applicable |
|  | 1. Collaborate with other professionals within the curative sector (such as social work, physiotherapists, occupational therapists, psychologists) to contribute to a solution to work-related problems | | Completely agree/agree/neutral/disagree/completely disagree/no opinion/not applicable |
|  | 1. Collaborating with occupational physicians on issues in the field of work and health (so called clinical work-integrating care) | | Completely agree/agree/neutral/disagree/completely disagree/no opinion/not applicable |
|  | 1. Provide information to the occupational physician or insurance physician (social security agency) | | Completely agree/agree/neutral/disagree/completely disagree/no opinion/not applicable |
|  | 1. Provide an information leaflet to the patient about legislation and regulations regarding absenteeism from work and disability due to illness | | Completely agree/agree/neutral/disagree/completely disagree/no opinion/not applicable |
|  |  | |  |
| *Questions about vision for the future* | | | |
| [Objective: ask for vision for the future] | | | |
| Toekomstvisie | Our goal is to create more awareness for ‘work’ within curative care. What would help you to pay more attention to work in your contact with patients? | | Availability of extra DBC ‘work’  Availability of a multidisciplinary care team with expertise in work  Availability of clinical occupational physician  Availability of outpatient clinic specialized in work  Other [text field]  [multiple answer options possible] |
| ToekomstvisieAnders |  | [if ‘other’ to the above question]  Can you explain this? | [Short free text] |
|  |  | | |
| *Conclusion* | | | |
| ToekomstBenader | May we approach you for a follow-up interview in response to this survey, or are you interested in further discussing with us about the position of work in curative care?  If we may contact you for an interview  please click here.  If you are interested in further discussing the content of this survey with us  Please click here  [i] Because of privacy legislation, a separate window will open in which you can directly email the coordinating researcher. As a result, your personal data will not be stored in the database of the survey. If this window does not open, you can also email directly to *** | | Mail to link in regard to privacy legislation and anonymous survey |
| Opmerkingen | Do you have any comments regarding this survey? | | [open comment box] |
|  |  | |  |
|  | Thank you very much for completing the survey. You can now click send. | |  |
