# Supplementary material for: The extent to which medical specialists provide Clinical Work-Integrating Care (CWIC) and their perceived role-responsibility: a mixed-methods study
Source: BMC Health Serv Res. 2025 Mar 27;25:448. doi: 10.1186/s12913-024-12137-y (PMC11948937; doi:10.1186/s12913-024-12137-y)
Supplement: Supplementary file 2 — Supplementary Material 2. [file 12913_2024_12137_MOESM2_ESM.docx]

**Interview guide – Attention to work in the consultation room**

**Introduction**

Welcome and thank you for joining today. I would like to begin with a brief introduction round and give a quick overview of the interview today before we get started.

***We already received some information about you, could you please provide a brief introduction to yourself?***

During the interview today, we would like to get a better understanding of the data from the survey that you and the other medical specialists completed earlier. Since this was an anonymous survey, we are unaware of your responses. The interview and the survey are based on a previous research regarding patients’ needs for clinical work-integrating care. We define this as paying attention to work in a clinical context, where the healthcare professional acts based on the notion that work and health are intertwined. There is a social trend to pay more attention to the factor ‘work’ in the clinic. Patients have indicated they also need this.

We would like to discuss three themes with you today:

Your opinion about clinical work-integrating care

Collaboration between clinical care and occupational healthcare specialists

Your opinion about your role in clinical work-integrating care following a needs analysis of the patient

We will record this interview, for which you have signed an informed consent.

**Do you have any questions about the informed consent or the interview itself?**

We will start the recording now.

**Topic: opinion medical specialist about clinical work-integrating care**

Clinical work-integrating care refers to: paying attention to work in a clinical context, where the healthcare professional acts based on the notion that work and health are intertwined.

**What do you consider clinical work-integrating care? Does this fall under your responsibilities?**

- To what extent do you include work as a factor in your treatment?

***Note****: We now know that some participants are already doing something special with this, such as a separate outpatient clinic. If applicable, ask about this.*

- To what extent is there room for clinical work-integrating care in current practice?
- Is there room for the factor work within current medical guidelines?
- Do you have an example of a situation in which the importance of continuing to work influenced treatment? How was this decided? Does shared decision making play a role in this?

**Do physicians in your profession see this the same way? How would you describe the norm within your profession?**

- In your experience, is the subject of clinical work-integrating care relevant in your profession?

- Do you discuss clinical work-integrating care with direct colleagues? How?

- To what extent is it a topic of discussion during your meetings?

**You have already given some examples of clinical work-integrating care. How did you get to the point where you included work as a factor?**

**OR: You indicate that it is not really your job to discuss work. But how would you feel about making returning to work a primary treatment goal?**

- For example, to get someone 100%/mostly back to work?

- Would you like to pay more attention to work?

- Do *you* feel competent to do this now? Can you explain?

**For rehabilitation physicians: What would you like to tell other medical specialists about involving work participation in treatment?**

**Topic: Collaboration between clinical care and occupational healthcare specialists**

We would now like to discuss collaboration between clinical care and occupational healthcare specialists in more detail, since the occupational physician is sometimes also involved in the patient’s guidance outside the hospital.

The survey shows that there is little contact between medical specialists and occupational physicians. This contact is usually initiated by the occupational physician in order to request medical data.

**How do you see the role of the occupational physician? Do you see added value in working (more closely/more often) with an occupational physician?**

- How would you like to use the occupational physician in your treatment?
- Should the patient, also in view of the previous topic, initiate contact with the occupational physician?
- What do you do if the patient does not have an occupational physician but does need one?

In order to achieve collaboration between clinical care and occupational healthcare specialists, we asked about a number of barriers in the survey. Some of the respondents indicated that they never had contact with an occupational physician or insurance physician, that they were unaware of what tasks an occupational physician exactly has, or that they were unsure about privacy legislation and legal consequences.

**What is this like for you?**

- What is your opinion about the separation between occupational health care and clinical care?
- What do you know about the patient confidentiality of the occupational physician?
- How do you handle forwarding medical data (patient confidentiality)?

**What can we do to remove this barrier?**

**Topic: reflection of patient needs and role of the medical specialist**

Finally, we would like to discuss your role in clinical work-integrating care within the hospital based on a needs analysis of the patient. In different phases of care, patients struggle with different questions, which, of course, depend on the medical problem.

- Some patients are concerned about the influence of work on their health complaints and may benefit from direct prevention.
- Others have fallen out of work and would like to return to work in a sustainable way, and they may benefit from secondary prevention.
- Still others have permanently fallen out of work or have a permanently reduced capacity to work, they mainly struggle with questions about social benefits and what options they still have.

We will ask a number of questions about these aspects.

**Do you receive questions from your patients about health complaints caused by work? Do you have any examples of this? To what extent do you think you should actively ask about this? How can you help patients translate functional limitations into work?**

- Do you think you have to help with this, or do you consider it more the patient’s own responsibility?

- Or should the occupational physician provide this guidance? Or the general practitioner?

**To what extent do you consider guidance with stop working because of illness (either permanently or temporarily) as part of your work?**

- Do you ever have to indicate clear boundaries about what is and is not possible in connection with the patient’s health?
- Do you think a discussion about absenteeism and fitness for work has a place in the consulting room?
- Is emotional support part of that?

Patients need information about legislation and regulations in the Netherlands, especially if they are out of work for a long period of time, and expect help in finding the right information. Patients suggested receiving an information leaflet from the hospital. We also asked this in the survey. However, a large majority of the respondents indicated that they do not consider providing an information leaflet their responsibility.

**What is a suitable (acceptable) way to provide patients with this information?**

Several aspects of clinical work-integrating care have now been discussed, but we still have a few questions:

**At what point in the treatment would you like to pay attention to work?**

**What is the patient’s responsibility in this?**

**Is there still a role in clinical work-integrating care for other healthcare professionals in the hospital? If so, for whom and what?**

**Final questions**

We are at the end of the interview and still have two final questions:

**There are various solutions that can be devised from promoting clinical work-integrating care, do you have anything that would make a real contribution in your opinion?**

**What do you think of the term ‘clinical work-integrating care’ that we discussed? Do you still interpret this the same as at the beginning of the interview?**

**Do you have any other additions?**

**To thank**

Thank you for participating in this study, can we put you on the mailing list for the final article?
